# The role of RNA interference in the developmental separation of blood and lymphatic vasculature

**DOI:** 10.1186/2045-824X-6-9

**Published:** 2014-04-01

**Authors:** Sébastien Gauvrit, Josette Philippe, Matthieu Lesage, Marc Tjwa, Isabelle Godin, Stéphane Germain

**Affiliations:** 1 Collège de France, Center for Interdisciplinary Research in Biology (CIRB), 11, place Marcelin Berthelot, Paris F-75005, France; 2 CNRS UMR 7241, Paris F-75005, France; 3 INSERM U 1050, Paris F-75005, France; 4 ED 394: Physiologie et Physiopathologie, Université Pierre et Marie Curie, Paris F-75005, France; 5 Lab of Vascular Hematology/Angiogenesis, Goethe University Frankfurt, Frankfurt, Germany; 6 Institute for Transfusion Medicine, DRK Blutspendedienst, Goethe University Frankfurt, Frankfurt, Germany; 7 INSERM U1009, Villejuif F-94805, France; 8 Gustave Roussy, Villejuif F-94805, France; 9 Equipe labellisée Ligue contre le Cancer, Paris, France; 10 Department of Pathology, Saint-Louis Hospital, AP-HP, Paris F-75010, France

**Keywords:** Dicer, Lymphangiogenesis, Veino-lymphatic separation, Angiogenesis, RNA interference

## Abstract

**Background:**

Dicer is an RNase III enzyme that cleaves double stranded RNA and generates functional interfering RNAs that act as important regulators of gene and protein expression. Dicer plays an essential role during mouse development because the deletion of the *dicer* gene leads to embryonic death. In addition, dicer-dependent interfering RNAs regulate postnatal angiogenesis. However, the role of dicer is not yet fully elucidated during vascular development.

**Methods:**

In order to explore the functional roles of the RNA interference in vascular biology, we developed a new constitutive Cre/loxP-mediated inactivation of *dicer* in *tie2* expressing cells.

**Results:**

We show that cell-specific inactivation of *dicer* in *Tie2* expressing cells does not perturb early blood vessel development and patterning. *Tie2*-Cre; *dicer*^*fl/fl*^ mutant embryos do not show any blood vascular defects until embryonic day (E)12.5, a time at which hemorrhages and edema appear. Then, midgestational lethality occurs at E14.5 in mutant embryos. The developing lymphatic vessels of *dicer*-mutant embryos are filled with circulating red blood cells, revealing an impaired separation of blood and lymphatic vasculature.

**Conclusion:**

Thus, these results show that RNA interference perturbs neither vasculogenesis and developmental angiogenesis, nor lymphatic specification from venous endothelial cells but actually provides evidence for an epigenetic control of separation of blood and lymphatic vasculature.

## Background

RNA interference (RNAi) is a gene silencing pathway by which specific messenger RNAs (mRNAs) are either degraded or translationally suppressed [1]. It is mediated by microRNA (miRNA) or short interfering RNA (siRNA), both non coding RNAs of 20–22 nucleotides which are matured by the RNase Dicer and are involved in base pairing with target mRNAs. In mice, *dicer* is critical for early mouse development because its abrogation prevents the production of functional interfering RNAs resulting in embryonic lethality at E7.5 [2]. A second study reported death at E13.5 which was associated with angiogenesis defects [3] but both studies were unable to decipher the role of Dicer in specific vascular cell types. Conditional ablation of *dicer* developed to investigate its function in limb buds [4], in immune cells [5], and heart development [6] have suggested important roles of RNA interference in various biologic processes such as cell survival, proliferation, differentiation, and maintenance of cell function.

In angiogenesis, the role of Dicer-regulated miRNAs was further suggested in mice expressing a hypomorphic Dicer1 allele, which resulted in female infertility caused by corpus luteum insufficiency and defective ovarian angiogenesis [7]. In addition, Dicer has been shown to have multiple roles in vascular biology. Tamoxifen-inducible and smooth muscle cell (SMC)-specific deletion of Dicer achieved by Cre-Lox recombination showed that miRNAs are necessary for vascular smooth muscle growth, differentiation, and function [8,9]. Dicer-deficient mice exhibited a dramatic reduction in blood pressure due to significant loss of vascular contractile function and SMC contractile differentiation as well as vascular remodeling. This phenotype pointed to miRNAs as important mediators for the modulation of the VSMC phenotype by targeting transcription factors and the cytoskeleton, which acts as molecular switches for VSMC differentiation [10]. In these cells, the Mir143/145 gene cluster plays a major role in regulating the contractile phenotype and controling responses to various types of injury [11-13].

The reduction of endothelial miRNAs by inactivation of Dicer both *in vitro*[14] and *in vivo* using Cre-recombinase under the regulation of *tie2* promoter/enhancer or tamoxifen inducible expressed Cre-recombinase (Cre-ER^T2^) under the regulation of *vascular endothelial cadherin* promoter was shown to reduce postnatal angiogenic response to a variety of stimuli, including exogenous VEGF, tumors, limb ischemia, and wound healing [15]. *In vitro* studies demonstrated the presence of miRNAs in endothelial cells [16,17] and silencing of Dicer using short interfering (si)RNA in human endothelial cells resulted in impaired capillary-like structures and reduced cell growth [18-21]. The angiogenic properties of members of the mir 17–92 cluster have been extensively studied [15,22,23]. Also, miR-92a, miR-15a, miR-126 were identified to target mRNAs corresponding to several proangiogenic proteins, such as FGF2 and VEGF [22,24-28]. In addition, recent studies reported the role of miR-99b, miR-181a, and miR-181b in the differentiation of human embryonic stem cells to vascular endothelial cells [29]. In the vascular endothelium, recent findings have shown that miRNAs such as mir-210 orchestrate the response to hypoxia [30,31] and that down-regulation of Dicer under chronic hypoxia is an adaptive mechanism that serves to maintain the cellular hypoxic response through HIF-α and miRNA-dependent mechanisms [29]. Functional deficiency of Dicer in chronic hypoxia is relevant to both HIF-α isoforms and hypoxia-responsive/HIF target genes. The regulation of Prox1 by miR-181 further highlighted the contribution of RNA interference in the induction of lymphatic endothelium. Indeed, miR-181 is highly expressed in the blood vasculature, but significantly reduced in lymphatic endothelial cells, reciprocally to Prox1 expression [32].

However, whether Dicer could regulate angiogenesis, especially during development when hypoxia is a major stimulus remains largely unclear. There is still insufficient evidence for the involvement of RNA interference during the early stages of vascular cell development, and particularly in the control of endothelial arterial-, venous-, and lymphatic- fate specification. Here, we show that conditional inactivation of Dicer in mice expressing Cre recombinase under the control of the *tie2* promoter causes no major alterations in EC fates and differentiation but leads to unexpected functional and morphologic alterations in the separation of blood and lymphatic vasculature.

## Methods

### Mice

The experiments were performed in accordance with the guidelines of the French Ministry of Agriculture. This study conforms to the standards of INSERM (the French National Institute of Health) in accordance with European Union Council Directives (86/609/EEC). All experiments were performed blindly, meaning that the experimenter was blind to the mouse genotype.

Mice were backcrossed to the C57BL/6 J background for more than 10 generations.

*tie2-Cre:dicer*^*fl/+*^*(dicer*^*ΔEC/+*^) males were crossed with *dicer*^*fl/fl*^ females to generate embryos. The day of vaginal plug observation was considered as E0.5. Genotyping was performed on embryonic fragments using the following PCR primer pairs: Cre-R 5′-AACAGCATTGCTGTCACTTGGTCG-3′ and Cre-F 5′-ATTACCGGTCGATGCAACGAGTGA-3′ (product size: 350-bp); DicerF1 5′-CCTGACAGTGACGGTCCAAAG-3′ and DicerR1 5′-CATGACTCTTCAACTCAAACT-3′ (product sizes: 420-bp *dicer*^*Δ*^ allele and 351-bp wild-type *dicer* allele). ROSA26-R embryos were genotyped by PCR using three oligonucleotides: ROSA-1 5′-AAAGTCGCTCTGAGTTGTTAT-3′, ROSA-2 5′-GCGAAGAGTTTGTCCTCAACC-3′ and ROSA-3 5′-GGAGCGGGAGAAATGGATATG-3′. D*icer*^*fl/+*^ and *dicer*^*fl/fl*^ are thereafter designated as wild type (WT) embryos, *dicer*^*ΔEC/+*^ and *dicer*^*ΔEC/ΔEC*^ called heterozygous and mutant embryos respectively.

Efficient Cre recombinase-mediated excision of the floxed *dicer* allele was detected on PECAM^+^ endothelial cells from *dicer*^*ΔEC/+*^ and *dicer*^*ΔEC/ΔEC*^ embryos. Briefly, mouse tissues were incubated in 5 mL Dulbecco modified Eagle medium containing 200 U/mL collagenase I (Invitrogen) for 45 minutes at 37°C with occasional shaking followed by filtering through a 40-μm nylon mesh. The cells were then centrifuged for 5 minutes at 4°C, resuspended in Buffer 1 (0.1% bovine serum albumin, 2 mM EDTA pH 7.4 in phosphate-buffered saline) and incubated with anti rat immunoglobulin G-coated magnetic beads (Invitrogen) precoupled with rat anti–mouse platelet/endothelial cell adhesion molecule-1 (PECAM-1; MEC13.3, BD Pharmingen) for 30 minutes at 4°C. Beads were separated using a magnetic particle concentrator (Dynal MPC-S, Invitrogen). The beads were washed 5× with Buffer 1 and centrifuged for 5 minutes at 3400 *g*, and the supernatant removed as previously described [33]. PCR analysis was performed using primers DicerF1 and DicerDel 5′-CCTGAGCAAGGCAAGTCATTC-3′. The deletion allele produced a 471-bp PCR product whereas a wild-type allele resulted in a 1,300-bp product.

### X-Gal staining

Embryos were harvested at different stages and fixed in 4% formaldehyde for 10 min at RT, rinsed twice in 1X phosphate-buffered saline, and incubated overnight at 37°C in buffer containing PBS 1X, 0.1 M sodium phosphate (pH 7.3), 2 mM magnesium chloride, 0.02% NP-40, 0.01% sodium deoxycholate, 5 mM potassium ferricyanide, 5 mM potassium ferrocyanide, and 1 mg/ml X-gal (5-bromo-4-chloro-3-indoyl β-D-galactopyranoside).

### Histological analysis

Embryos were harvested, fixed in 4% paraformaldehyde overnight and embedded in paraffin. Histologic specimen of mouse tissue was stained with hematoxylin and eosin*.*

### Immunohistochemistry

Paraffin-embedded sections were deparaffinized, permeabilized, and incubated with goat polyclonal anti-VEGFR-3 (1:100, R&D Systems) or anti VEGFR-2 (1:100, R&D Systems) followed by biotin-streptavidin-HRP amplification using the Vectastain-ABC kit (Vector Lab), and post-stained with eosin.

For whole-mount staining, tissues were fixed overnight in 4% PFA and blocked overnight in blocking buffer (PBS, 5% goat serum, 0.3% Triton X-100, and 0.2% BSA). Tissues were incubated overnight at 4°C with biotinylated anti–mouse LYVE-1 (1:100, R&D Systems) or PECAM-1 (1:100, BD Biosciences) in blocking buffer followed by biotin-streptavidin-HRP amplification using the Vectastain-ABC kit.

## Results

**Figure 1:**
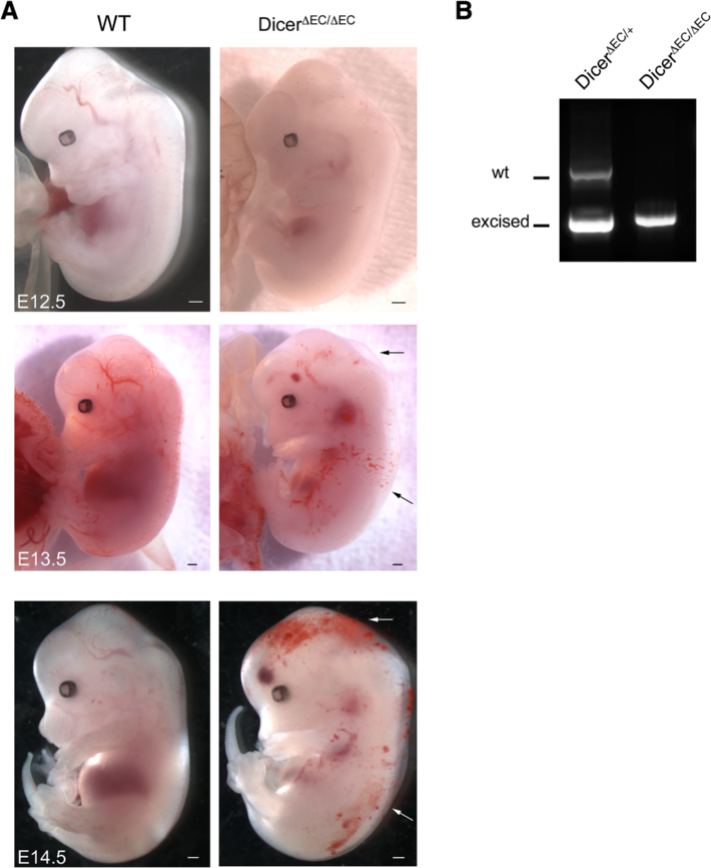
**Conditional deletion of *dicer *gene leads to hemorrhage and edema. A)** Whole-mount view of WT and *dicer*^*ΔEC/ΔEC*^ embryos from E12.5 to E14.5. Hemorrhagic regions and edema are indicated (arrows). Scale Bar: 500 μm. **B)** PCR genotyping analysis of PECAM^+^ endothelial cells from E13.5 *dicer*^*ΔEC/+*^ and *dicer*^*ΔEC/ΔEC*^ embryos. Detection of Cre and *dicer* fragments (floxed, excised and WT) are presented.

To bypass the early embryonic lethality of *dicer*-null mice [2], we developed a new Cre-loxP-mediated conditional deletion of *dicer* in tie2-expressing cells in order to investigate its role in vascular development. To this end, we crossed *dicer*-floxed mice (*dicer*^*fl/fl*^) [4] with *tie2*-Cre transgenic mice [4,34]. The resulting heterozygous double transgenic mice (*dicer*^*ΔEC/+*^) were viable. Intercrosses of *dicer*^*ΔEC/+*^ male with *dicer*^*fl/fl*^ females yielded no *dicer*^*ΔEC/ΔEC*^ pups out of 293 viable offspring at birth (see Table 1). These data suggested that mice bearing *dicer* gene deficiency in *tie2*-expressing cells do not survive embryogenesis. To determine when the *dicer*^*ΔEC/ΔEC*^ mice died, embryos were examined from E10.5 to birth. Embryos were removed and embryonic DNA was analyzed for homo- or heterogeneity of the floxed allele. This genotype was then correlated with the viability of the embryo. Mendelian ratios were observed from E10.5 to E13.5 (see Table 1). Mutant embryos from E10.5 to E11.5 were macroscopically indistinguishable from the control littermates. At later stages, E12.5 onwards, macroscopic examination revealed the presence of hemorrhages and edema in mutant embryos that increased in size and number with age (Figure 1A). Genotyping PECAM^+^ endothelial cells showed efficient *dicer* inactivation in E13.5 *dicer*^*ΔEC/ΔEC*^ embryos compared to E13.5 *dicer*^*ΔEC/+*^ embryos here used as controls (Figure 1B).

**Table 1:** **Genotype analysis in percentages of live embryos resulting from the cross of a *dicer***^***Δ/+ ***^**male with a *dicer***^***fl/fl ***^**female**

	**WT**	***dicer***^***ΔEC/+***^	***dicer***^***ΔEC/ΔEC***^
Expected ratios	50%	25%	25%
E10.5 n = 119	47.1%	28.5%	24.4%
E11.5 n = 49	49%	30.8%	20.4%
E12.5 n = 31	37.5%	38.7%	23.8%
E13.5 n = 90	36.6%	26.7%	36.7%
E14.5 n = 29	34.5%	23.8%	9.5%
E15.5 n = 4	50%	50%	0%
P14 n = 293	63.9%	36.1%	0%

**Figure 2:**
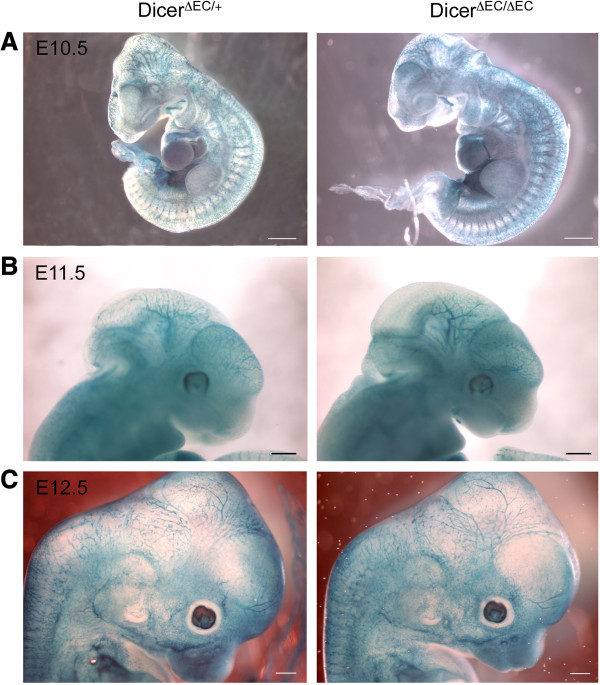
**Mutant embryos do not display vascular defects. A-C)** Whole-mount view of X-Gal staining of *dicer*^*ΔEC/+*^:R26/+ embryos and *dicer*^*ΔEC/ΔEC*^:R26/+ embryos from E10.5 to E12.5. Scale Bar: 500 μm. The vascular network is identical in mutant and control embryos (n = 3 for each condition).

**Figure 3:**
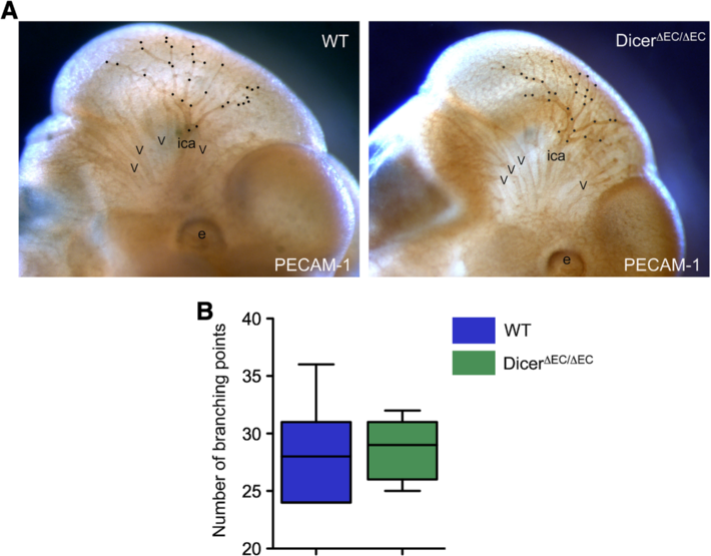
**Blood vessel patterning normally occurs in mutant embryos. A)** Whole-mount immunohistochemical staining by anti-PECAM-1 antibody on E11.5 embryos. Scale Bar: 500 μm. **B)** Branchpoints quantification (means ± SEM) of the internal carotid artery (ica) on E11.5 embryos. The number of branchpoints is similar in WT and *dicer*^*ΔEC/ΔEC*^ embryos (ica; dots represent arterial branchpoints; e, eye; v, veins). (WT n = 10, *dicer*^*ΔEC/ΔEC*^ n = 5).

Recombination was also monitored in *tie2*-expressing cells using the ROSA26 (R26) reporter line [35]. We then crossed the *dicer*^*ΔEC/+*^ males with homozygous *dicer*^*fl/fl*^:R26/R26 females to generate *dicer*^*ΔEC/+*^:R26/+ (heterozygous, here as a control) and *dicer*^*ΔEC/ΔEC*^:R26/+ (mutant) triple transgenic embryos. As indicated by whole-mount X-Gal staining, the recombination was efficient in blood endothelial cells thereby allowing us to compare the pattern of the vascular network in mutant and control embryos using LacZ staining (Figure 2). Between E10.5 and E12.5, *dicer*^*ΔEC/ΔEC*^ embryos did not display obvious blood vascular defects: avascular regions were not observed in control embryos. X-Gal-stained blood vessels formed properly and vascular density was comparable in both control and mutant embryos (Figure 2). Whole-mount staining using an anti-PECAM antibody confirmed these observations as reported in Figure 3A showing that vascular patterning of blood vessels was comparable to controls in E11.5 *dicer*^*ΔEC/ΔEC*^ embryos. In order to study the development of the blood vessel network in greater details, branchpoints of the cranial vascular network (internal carotid artery) were quantified on E11.5 embryos. The number of branchpoints in the internal carotid artery was not statistically different in *dicer*^*ΔEC/ΔEC*^ embryos compared to WT embryos (Figure 3B).

**Figure 4:**
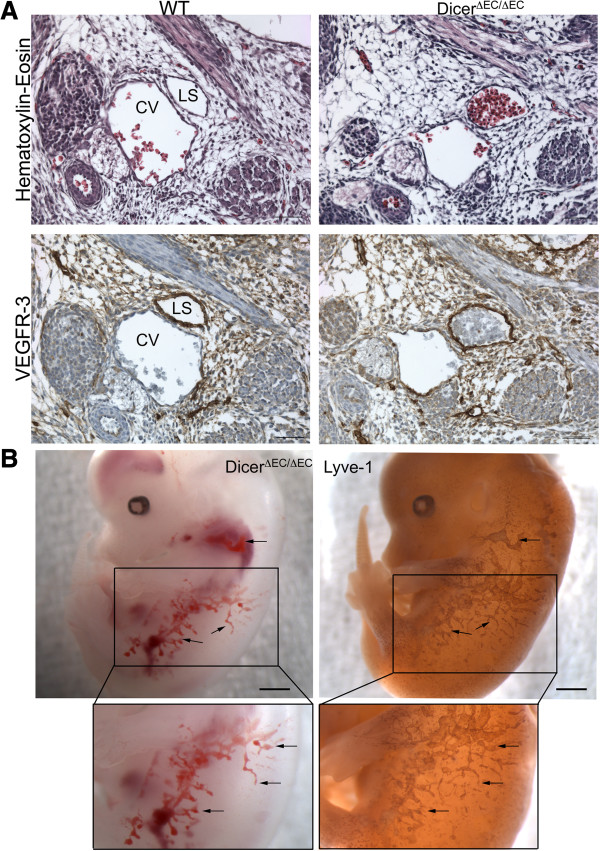
**Mutant embryos present blood-filled lymphatics. A)** Histological analysis of E13.5 WT and *dicer*^*ΔEC/ΔEC*^ embryos. *dicer*^*ΔEC/ΔEC*^ embryos display blood-filled structures contrary to WT embryos as revealed after hematoxylin/eosin staining (HE) (upper panel). Immunostaining with the lymphatic marker VEGFR-3 confirmed the lymphatic identity of the blood filled structures (lower panel). Cv: cardinal vein, ls: lymph sac. Scale bar: 50 μm. (n = 2 for each condition). **B)** Whole-mount view of a E14.5 *dicer*^*ΔEC/ΔEC*^ embryo after dissection (Left panel). Hemorrhages are indicated by Δarrows. Immunohistochemical staining by anti-LYVE-1 antibody on the same mutant embryo (Right panel). Scale Bar: 500 μm. Higher magnification of the *dicer*^*ΔEC/ΔEC*^ embryo after dissection and after LYVE-1 staining respectively (Lower panel). There is a complete overlap between hemorrhages and LYVE-1 staining indicating blood-filled lymphatics in the *dicer*^*ΔEC/ΔEC*^ embryo. (n = 2).

Between E12.5 and E14.5, extensive edema gradually progressed on the back of the embryos and was sometimes filled with blood cells in *dicer*^*ΔEC/ΔEC*^ embryos which were all dead at E14.5 (Figure 1 and Table 1). This also phenocopies the effects observed upon genetic deletion of Prospero homeobox 1 (*prox-1*) [36], Src homology domain-containing leukocyte protein-76 (*slp-76)*[37] or C-type lectin-like receptor 2 (*clec-2*) [38]. All show impaired lymphatic vessel development and die *in utero* with severe edema and hemorrhages. To establish whether *dicer*^*ΔEC/ΔEC*^ embryos also present defects in lymphatic vessels development, we examined transverse sections of mutant embryos. At E13.5, we never observed any disruption of the main blood vessels i.e. the thoracic aorta or the cardinal vein in *dicer*^*ΔEC/ΔEC*^ embryos (Additional file 1: Figure S1 and Figure 4). The lymph sacs, the first lymphatic structure that emerges from the cardinal vein [39] during development, also appeared normal (Additional file 2: Figure S2). At E13.5 however, in contrast to control embryos, these lymph sacs were filled with blood cells in *dicer*^*ΔEC/ΔEC*^ embryos (Figure 4A). The lymphatic identity of the blood-filled structures was confirmed by the expression of lymphatic markers VEGFR-3 (Figure 4A) and PROX-1 (Additional file 3: Figure S3). Moreover, LYVE-1 whole-mount immunostaining evidenced a complete overlap between blood-filled structures and the lymphatic vasculature in E14.5 mutant embryos (Figure 4B), confirming the blood-filled lymphatics phenotype.

Altogether, these data indicate that *dicer* inactivation in *tie2* expressing cells leads to embryonic lethality at E14.5, and to a failure in the separation of lymphatic vessels during embryonic angiogenesis.

## Discussion

Here, using Cre/loxP-mediated inactivation of *dicer* in *tie2*-expressing cells, we demonstrate for the first time that embryonic venous-lymphatic separation is submitted to epigenetic control by RNA interference. Previous studies using a similar approach of conditional *dicer* deficiency using *tie2*-Cre and ve-cadherin-CRE-ERT2 have reported reduced postnatal angiogenesis but no developmental defects [15]. The likely explanation for this discrepancy probably relies on the use of a different dicer-floxed mouse leading to the presence of residual Dicer protein levels in *tie2*-Cre:*dicer*^*fl/fl*^ endothelial cells, reflecting an incomplete excision of the *dicer* allele [15]. Thus, these mice were hypomorphic for *dicer* in ECs and *tie2*-Cre:*dicer*^*fl/fl*^ newborn litters were overtly normal and indistinguishable from their littermate controls. In contrast, in the present study, efficient *dicer* inactivation was evidenced in PECAM^+^ endothelial cells which showed complete excision of *dicer* in *dicer*^*ΔEC/ΔEC*^ embryos. The present study thus shows that *dicer* gene deletion in *Tie2* expressing cells leads to embryonic lethality at E14.5. Mutant embryos, which display hemorrhages and edema, showed blood-filled lymphatics without evident angiogenesis defects at early stages.

We here used the well-documented *tie2-*Cre transgenic mice that express Cre in a pan-endothelial fashion for vascular endothelial targeting [34]. With the Rosa26 reporter line, we showed recombination in lymphatic vessels (Additional file 4: Figure S4). Using the same *tie2-*Cre ROSA26 strain, Srinivasan et al. demonstrates that at E11.5, Prox1^+^ endothelial cells in the anterior cardinal vein and those budding from it were *lacZ*^+^. Similarly, all E13.5 and E14.5 Prox1^+^ endothelial cells in the lymph sacs were *lacZ*^+^[40]. Nevertheless, it should be noted that it has also been reported that *tie2-*Cre transgenic mice express *Cre* in blood island progenitors [41,42]. Recent studies have highlighted the role of hematopoietic cells during the process of separation between the venous and the lymphatic vasculature. It has been shown that podoplanin, a transmembrane protein expressed on lymphatic endothelial cells, engages the platelet receptor CLEC-2 leading to Syk-Slp-76-dependent platelet activation [43]. Deletion of these genes leads to aberrant vascular connection between blood and lymphatic vessels. Similar lymphovenous connections were also observed in mice deficient for the homeodomain transcription factor Meis1 (myeloid ecotropic viral integration site 1) which completely lack megakaryocyte/platelets and for the transcription factor Runx1 which lack hematopoietic stem cells [40,44]. It should also be noted that runx1 mutant embryos, which lack platelets, present hemorrhages in the brain [45], which could also be observed in some *dicer*^*ΔEC/ΔEC*^ embryos. Because platelets also act to maintain vascular integrity and as the brain and lungs are more susceptible to haemorrhage in a mouse model of acute severe thrombocytopenia induced by platelet depletion [46], these hemorrhages most likely occur secondary to the lack of platelets. These data showed that platelets are required during embryonic lymphangiogenesis for the separation of the nascent lymphatic vasculature from blood vessels [47,48]. However, recent studies by Yang et al. [49] and Hägerling et al. [50] have disproved a direct involment of platelets in the emergence of the first jugular lymph sacs. Podoplanin expression only starts after lymphatic endothelial cells leave the cardinal vein suggesting that platelets have a role restricted to the region where lymphatics and blood vessels coalesce, in the lymphovenous valves. Nevertheless, the presence of blood cells in lymphatic vessels may also indicate an incomplete separation of blood and lymph vessel, but could also result from *de novo* connections of previously separated blood and lymph vessels. Recently, Hess et al. proved that platelets interact with lymphatic endothelium valves specifically at the thoracic duct-subclavian vein junction [51]. Blood-filled lymphatics arise due to backfilling of the lymphatic vascular network from this site either due to a lymphovenous valve defect or due to a platelet aggregation defect. We therefore looked at the thoracic duct-subclavian vein junction and we determined that the lymphovenous valves appears normal (Additional file 5: Figure S5) suggesting a defect in platelet aggregation.

We therefore sought to decipher whether perturbing *dicer* expression in megakaryocytes could also reproduce a blood-filled lymphatic phenotype during development by generating *pf4*-cre:*dicer*^*fl/fl*^ mice. *Pf4*-cre express Cre-recombinase in the megakaryocytic lineage as previously shown [52] and are a useful tool to study megakaryopoiesis, and platelet function. These mice were born at normal mendelian ratio and the separation of the lymphatic vasculature from the blood vessels was not disrupted during development (Additional file 6: Figure S6 and Table 2). Recombination was observed in liver megakaryocytes before venous-lymphatic separation, as soon as E11.5 (data not shown) and persisted at E16.5 (Additional file 4: Figure S4B). However, the *pf4*-Cre transgene is also partially expressed in other hematopoietic lineages and the recombination pattern during early embryogenesis is not clear [53]. A megakaryocyte specific promoter that could allow earlier deletion might be useful but does not exist.

**Table 2:** **Genotype analysis in percentages of live pups resulting from the cross of a *pf4*-cre:** ***dicer***^***Δ/+ ***^**male with a *dicer***^***fl/fl ***^**female**

	**WT**	***pf4*-cre** ***:dicer***^***Δ/+***^	***pf4*-cre: *dicer***^***Δ/Δ***^
Expected ratios	50%	25%	25%
P14 n = 40	45%	30%	25%

Also, cells from the myeloid lineage play a critical role in this separation. Abnormal infiltration of a specific monocyte population in *syk*-deficient mice leads to lymphatic hyperplasia, vessel dilation and blood-lymphatic shunts [54]. Tie2 is expressed in the early yolk sac mesoderm suggesting that recombination may occur in hematopoietic cells [55]. The use of a more endothelial specific strains such as *ve-cadherin*-CRE-ERT2 [56] or *pdgfb*-CRE-ERT2 [57] would also be very useful for understanding the specific role of Dicer in the endothelium. However, the CRE activation is tamoxifen-dependent making these models more suitable for postnatal angiogenesis as recombination at a precise embryonic time point might be somewhat difficult to achieve in a very reproducible manner.

MicroRNAs are involved in many aspects of physiological and malignant hematopoiesis but surprisingly, no existing studies have focused on the role of *dicer* during hematopoietic development. However, *dicer* invalidation in adult has been described. Buza-Vidas et al. showed that *dicer* is required during erythroid lineage differentiation [58]. It was also suggested that Dicer is involved in the regulation of the hematopoietic stem cell niche as well as the regulation of hematopoietic stem cell number [59,60]. The blood filled phenotype that we observed could result from either a defect of hematopoiesis or a volume expansion of the blood stream indirectly affecting lymphatic development. We therefore believe that further experiments, outside of the scope of the present manuscript, will be needed to determine precisely whether hematopoiesis is modulated in *dicer*^*ΔEC/ΔEC*^ embryos and to fully decipher the cellular and molecular mechanisms responsible for the blood-filled lymphatic phenotype in these mice.

## Conclusion

Taken together, these results show a new role for RNA interference in epigenetic control of embryonic venous-lymphatic separation and provide a knowledge base for further investigations to validate functional roles for microRNAs.

## Abbreviations

CLEC-2: C-type lectin-like receptor 2; Cv: Cardinal vein; E: Embryonic day; Ica: Internal carotid artery; Ls: Lymph sac; miRNA: microRNA; mRNA: Messenger RNA; Pecam-1: Platelet endothelial cell adhesion molecule 1; siRNA: Short interfering RNA; Vegfr-3: Vascular endothelial growth factor receptor 3; WT: Wild type.

## Competing interests

The authors declare that they have no competing interests.

## Authors’ contribution

SGe, SGa designed experiments. SGa, JP and ML performed experiments. SGe, SGa, IG and MT wrote the paper. All authors read and approved the final manuscript.

## Supplementary Material

Additional file 1: Figure 1 Histological analysis of E13.5 thoracic aorta in WT and *dicer*^*ΔEC/ΔEC*^ embryos. Immunostaining with VEGFR-2 confirmed a normal patterning of the thoracic aorta of *dicer*^*ΔEC/ΔEC*^ embryos. Scale Bar: 2 μm. (n = 3). Click here for file

Additional file 2: Figure 2 Mutant embryos do not present lymph sacs defect. Whole-mount view of E12.5 WT and *dicer*^*ΔEC/ΔEC*^ embryos after LYVE-1 staining. The mutant embryo do not show a lymph sac defect. (n = 3 for each condition). Click here for file

Additional file 3: Figure 3 Prox1 expression on transversal sections of E13.5 WT and *dicer*^*ΔEC/ΔEC*^ embryos (n = 2 for each condition). Prox1 expression is maintained in lymphatics vessels in mutant embryos (upper panel), and the number of Prox1 expressing cells is similar in WT and *dicer*^*ΔEC/ΔEC*^ embryos (lower panel). Click here for file

Additional file 4: Figure 4 Whole-mount view of X-Gal staining of *dicer*^*ΔEC/ΔEC*^:R26/+ embryos at E13.5. Mutant embryo present recombination in lymphatic vessels (indicated by arrows). (n = 5). Click here for file

Additional file 5: Figure 5 Histological analysis of E13.5 lymphovenous valves in WT and *dicer*^*ΔEC/ΔEC*^ embryos (indicated by arrows). Immunostaining with VEGFR-3 showed a normal patterning and morphology of the lymphovenous valves of *dicer*^*ΔEC/ΔEC*^ embryos. Scale Bar: 2 μm. (n = 2 for each condition). Click here for file

Additional file 6: Figure 6 Conditional deletion of *dicer* in megakaryocytes does not lead to embryonic lethality. A) Whole-mount view of WT and *pf4*-cre:*dicer*^*Δ/Δ*^ embryos at E16.5. Mutant embryos do not present any obvious phenotype. B) Whole-mount view of X-Gal staining of a *pf4*-cre:*dicer*^*Δ/Δ*^ liver at E16.5 (Left panel). Histological analysis of the same E16.5 liver (Right panel). Recombination occurs in typical large megakaryocytes in the liver. (n = 3). Click here for file
