# Supplementary figures and images for: The role of RNA interference in the developmental separation of blood and lymphatic vasculature
Source: Vasc Cell. 2014 Apr 1;6:9. doi: 10.1186/2045-824X-6-9 (PMC4021977; doi:10.1186/2045-824X-6-9)

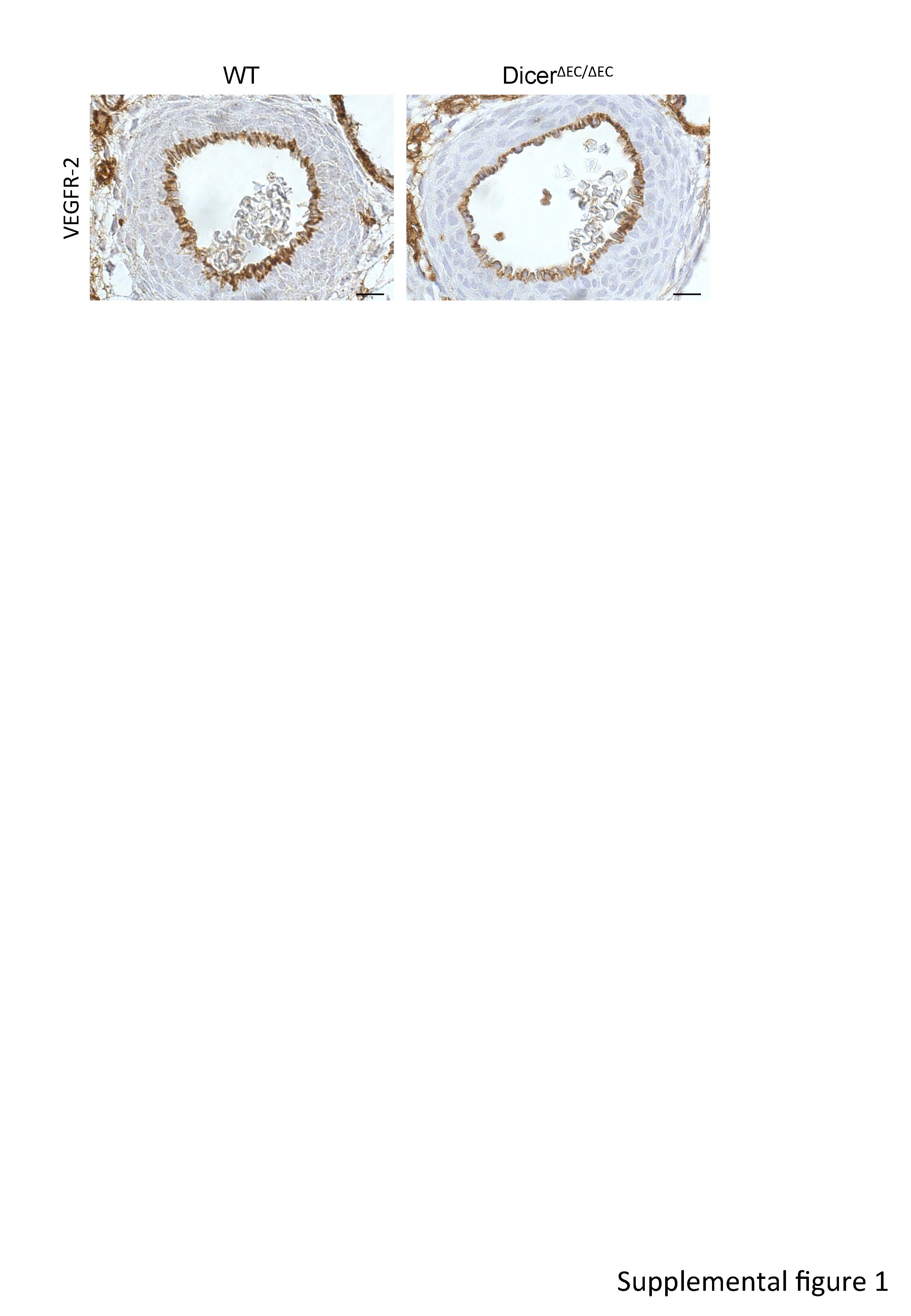

Supplement: Additional file 1: Figure 1 — Histological analysis of E13.5 thoracic aorta in WT and dicerΔEC/ΔEC embryos. Immunostaining with VEGFR-2 confirmed a normal patterning of the thoracic aorta of dicerΔEC/ΔEC embryos. Scale Bar: 2 μm. (n = 3). [file 2045-824X-6-9-S1.jpeg]

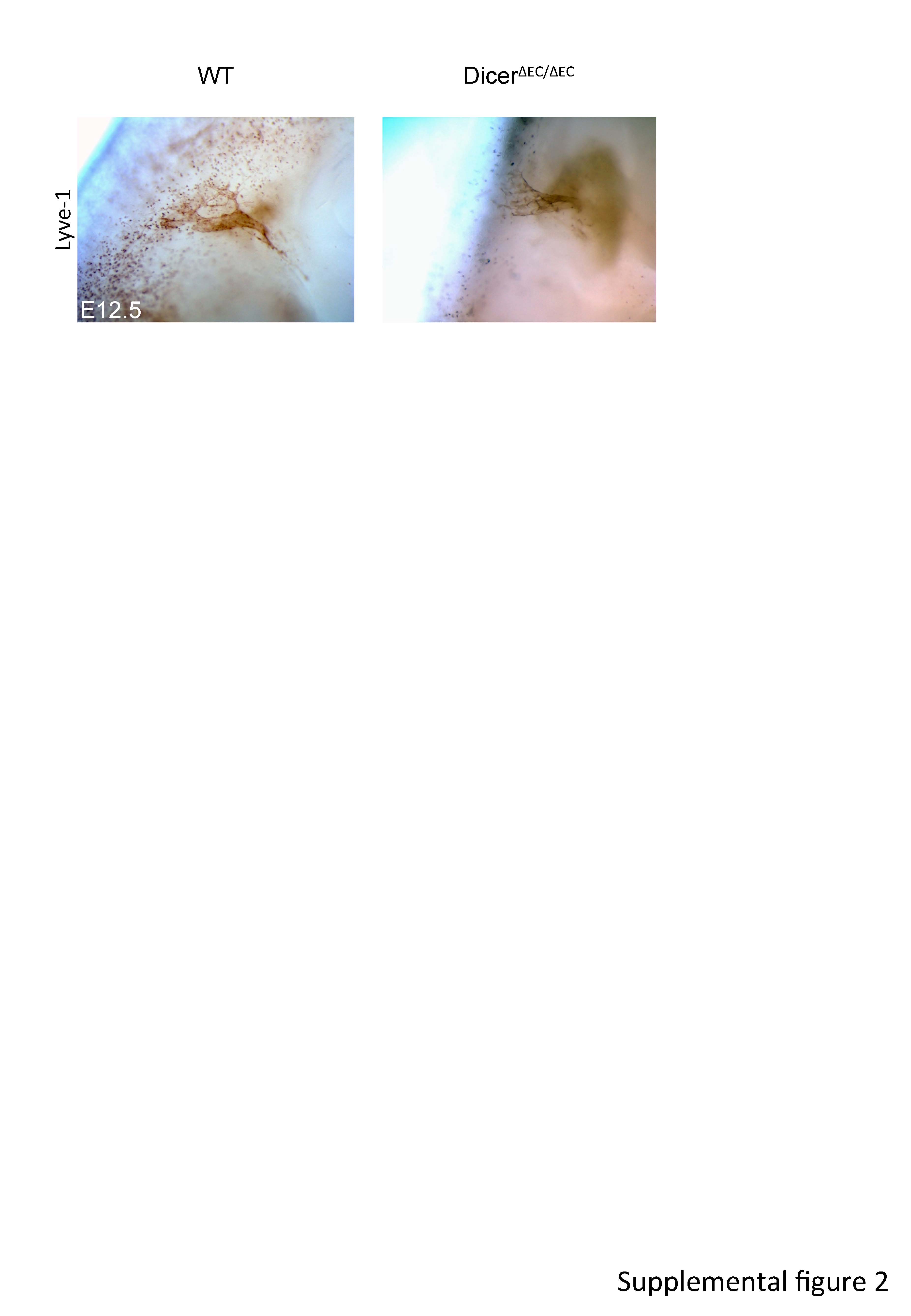

Supplement: Additional file 2: Figure 2 — Mutant embryos do not present lymph sacs defect. Whole-mount view of E12.5 WT and dicerΔEC/ΔEC embryos after LYVE-1 staining. The mutant embryo do not show a lymph sac defect. (n = 3 for each condition). [file 2045-824X-6-9-S2.jpeg]

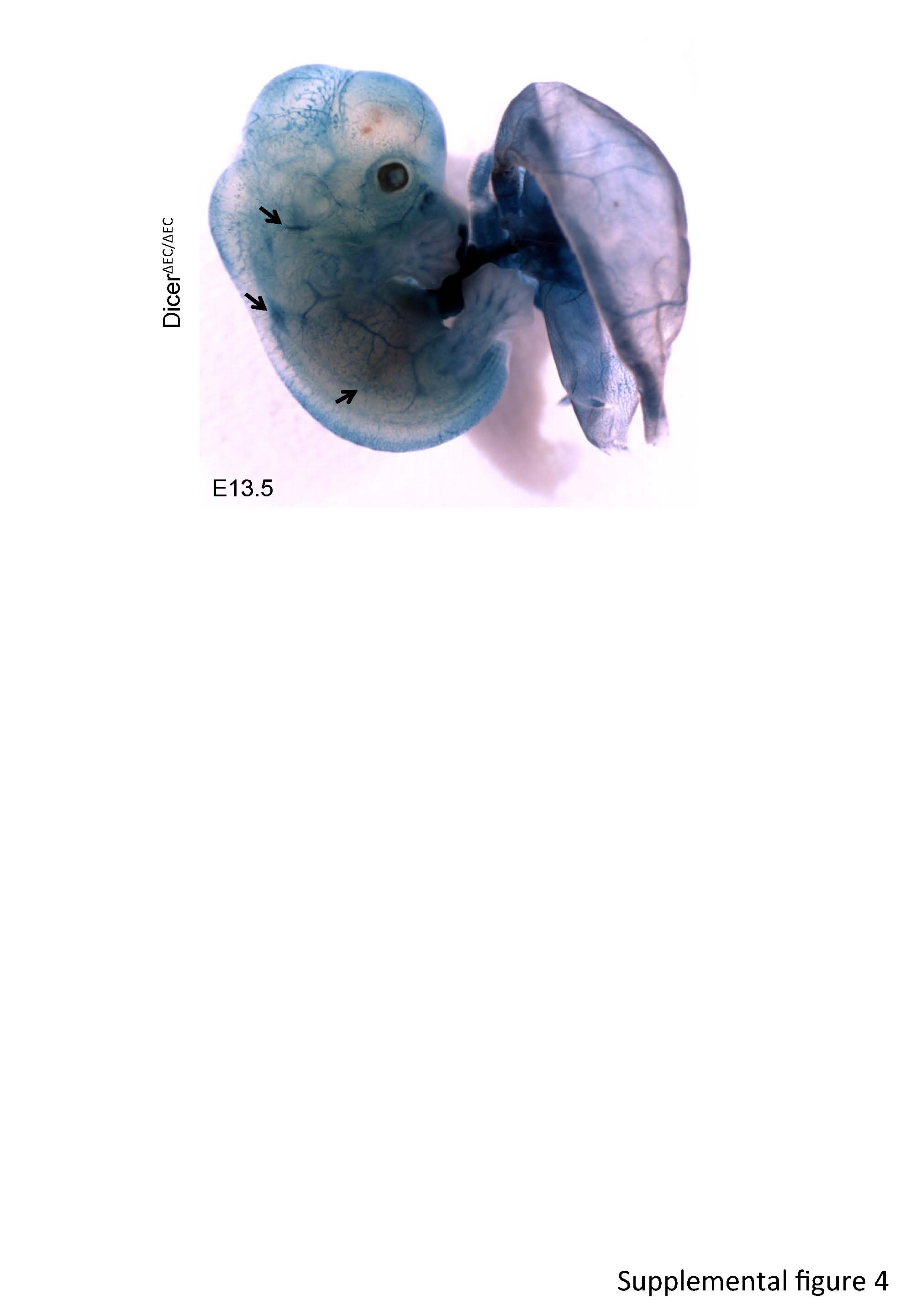

Supplement: Additional file 4: Figure 4 — Whole-mount view of X-Gal staining of dicerΔEC/ΔEC:R26/+ embryos at E13.5. Mutant embryo present recombination in lymphatic vessels (indicated by arrows). (n = 5). [file 2045-824X-6-9-S4.jpeg]

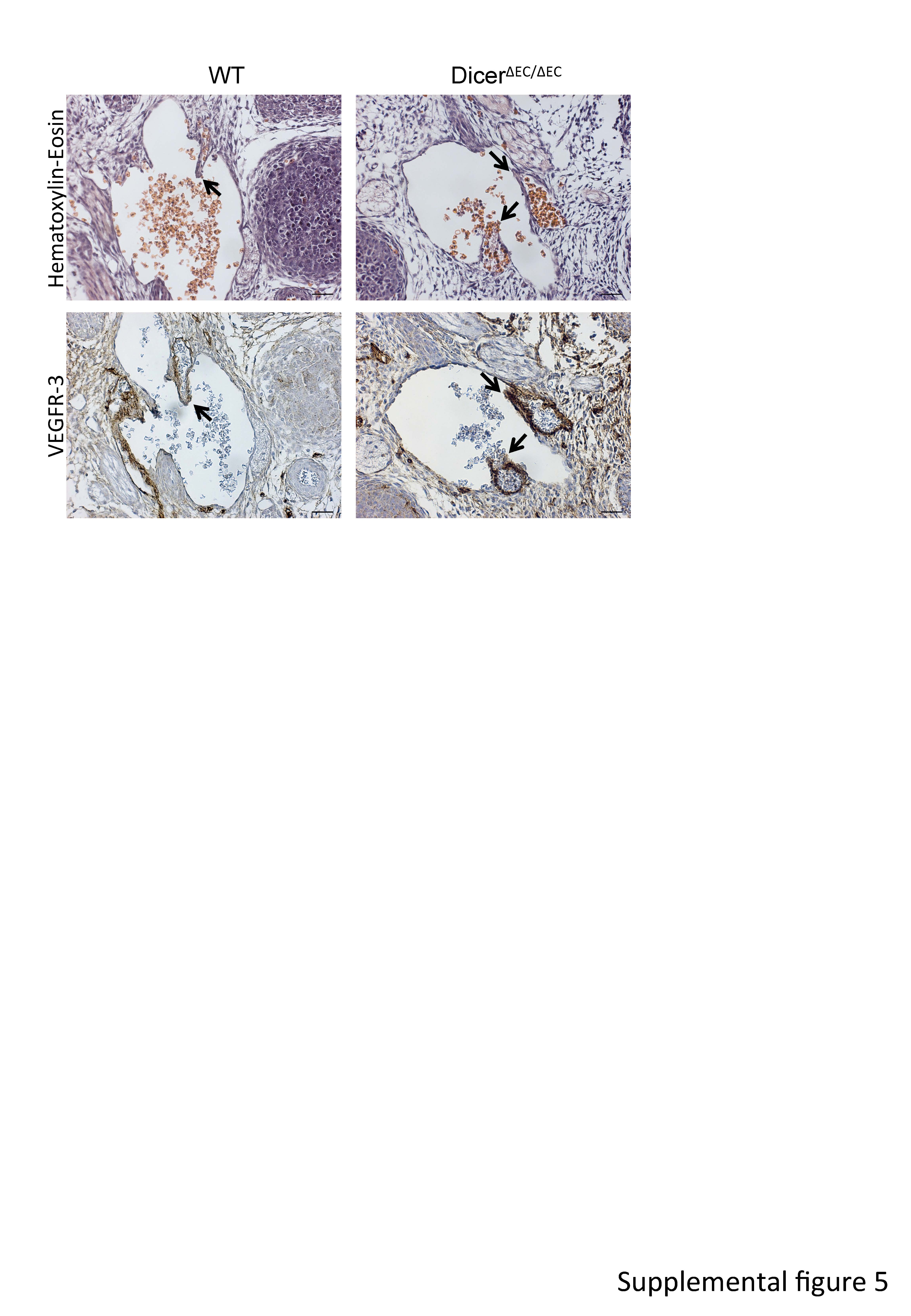

Supplement: Additional file 5: Figure 5 — Histological analysis of E13.5 lymphovenous valves in WT and dicerΔEC/ΔEC embryos (indicated by arrows). Immunostaining with VEGFR-3 showed a normal patterning and morphology of the lymphovenous valves of dicerΔEC/ΔEC embryos. Scale Bar: 2 μm. (n = 2 for each condition). [file 2045-824X-6-9-S5.jpeg]

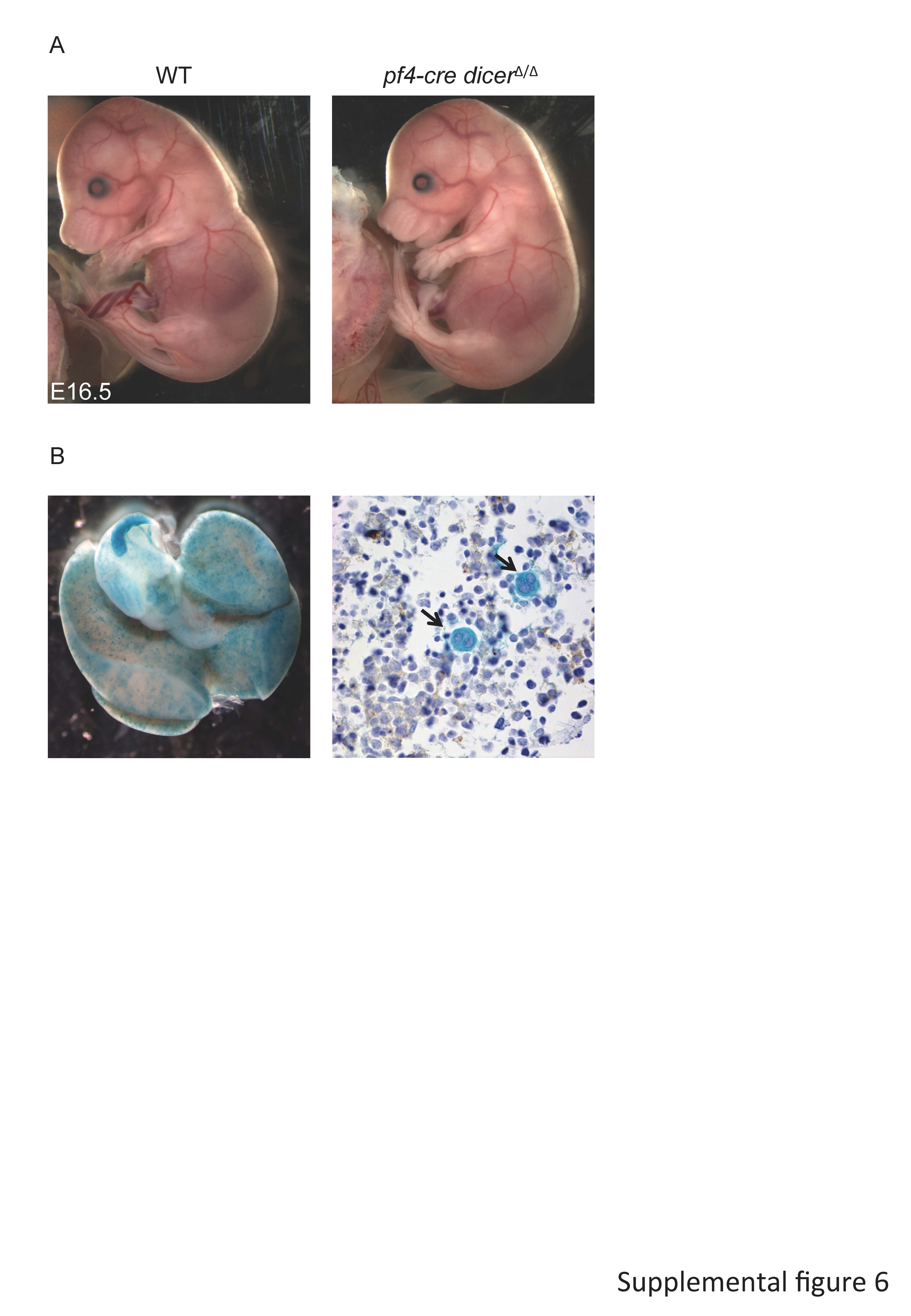

Supplement: Additional file 6: Figure 6 — Conditional deletion of dicer in megakaryocytes does not lead to embryonic lethality. A) Whole-mount view of WT and pf4-cre:dicerΔ/Δ embryos at E16.5. Mutant embryos do not present any obvious phenotype. B) Whole-mount view of X-Gal staining of a pf4-cre:dicerΔ/Δ liver at E16.5 (Left panel). Histological analysis of the same E16.5 liver (Right panel). Recombination occurs in typical large megakaryocytes in the liver. (n = 3). [file 2045-824X-6-9-S6.jpeg]
